# Case Report: Fatal Neurotoxicity Following Resmethrin Poisoning in a Child

**DOI:** 10.3389/fped.2021.746950

**Published:** 2021-11-19

**Authors:** Lilin Huang, Shumei Peng, Ronghan Li, Dongping Huang, Danyu Xie

**Affiliations:** Department of Pediatrics, Guangdong Women and Children Hospital, Guangzhou, China

**Keywords:** resmethrin poisoning, neurotoxicity, neuroimaging, child, pyrethroid

## Abstract

Resmethrin, a type I pyrethroid insecticide, can activate sodium channels, causing neurotoxicity in both mammals and insects. Possible routes of poisoning include inhalation, dermal contact and ingestion. There are no specific symptoms for resmethrin poisoning. Until now, no antidote has been available for resmethrin. Resmethrin poisoning is rarely reported in children. Here, we report a fatal case of resmethrin poisoning that might have been caused by accidental ingestion by a 26-month-old child. He presented with neurotoxic symptoms that included vomiting, recurrent seizures, and coma. The cranial CT showed extensive lesions of low intensity in the bilateral white matter, thalamus, brainstem, and cerebellum. Lumbar punctures showed increased intracranial pressure (ICP > 25 mmHg). Cerebrospinal fluid (CSF) tests revealed that protein was elevated to 289.2 mg/dL without pleocytosis. Resmethrin was detected in his blood by liquid chromatography-mass spectrometry, which confirmed the diagnosis of resmethrin poisoning. The child developed brain stem herniation and then was declared brain dead at the 77th h after admission. Resmethrin poisoning can be fatal, and it requires immediate diagnosis and treatment. Previous studies reported that cranial CT and CSF analyses were all normal in patients with pyrethroid poisoning. This case might extend the knowledge of neuroimaging and CSF analysis in children with resmethrin poisoning.

## Background

Pyrethroids are widely used as insecticides with low mammalian toxicity (1). However, there are still occasional pyrethroid poisoning reports. Pyrethroids divide into Type I (tremors) and Type II (choreoathetosis with salivation) relating to different intoxication syndrome, which both show neurotoxicity by activating sodium channels in neurons (2). The manifestations of pyrethroid poisoning are unspecific and can be abnormal sensations, irritative symptoms of the skin, fatal neurotoxicity, lung edema, and arrhythmia (3–5). Resmethrin is the first modern type I pyrethroid insecticide which is less toxic to mammals than natural pyrethrins. However, its insecticidal activity was equal to or greater than that of other pyrethrin I insecticides (6). In fact, resmethrin is a mixture of four isomers, including bioresmethrin, the ester of 1R, trans-chrysanthemic acid, cismethrin, the ester of 1R, and cis-chrysanthemic acid (6). Among these isomers, cismethrin showed intensive intracerebral toxicity to mice with an LD50 of 0.6 (mg/kg) (6). There were a few reports of pyrethroid poisoning in children who all had good prognoses (4, 7, 8). The cranial CT and cerebrospinal fluid (CSF) analysis were normal in these patients. Data about the resmethrin poisoning is rare. To our knowledge, our case is the first to describe the abnormal results of cranial CT and CSF analysis resulting from resmethrin poisoning in children. Extensive lesions in the brain and elevated protein in CSF might indicate the severity of illness and poor prognosis in our study. The clinical course, diagnosis, treatment, and prognosis of this fatal case were described and discussed.

## Case Report

**Figure 1:**
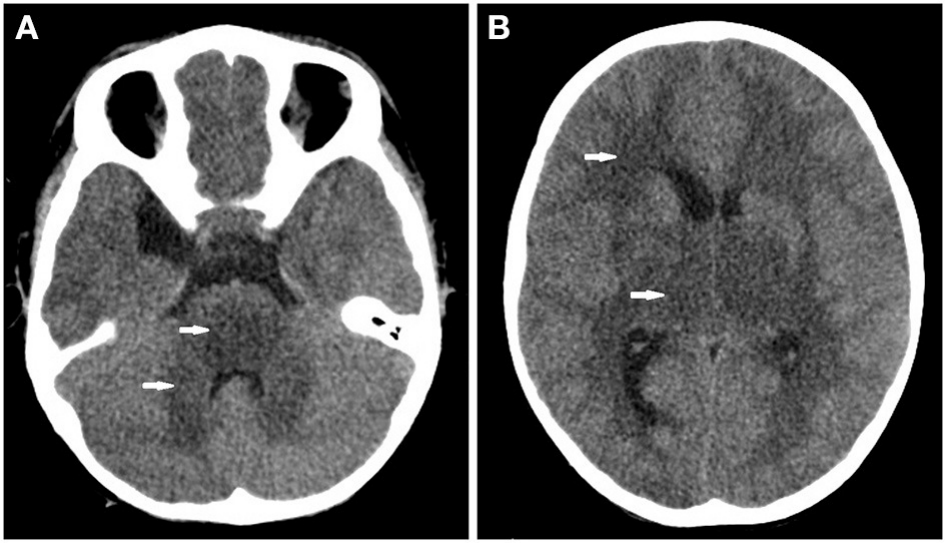
Cranial CT found **(A)** extensive lesion of low intensity in the pons and cerebellum (white arrow) and **(B)** the bilateral white matter and thalamus (white arrow).

A previously healthy 26-month-old child was admitted to our hospital for a 12-min history of recurrent tonic-clonic seizures and depression of consciousness. He showed a transient tremor 11 h prior to the seizure. He presented a mild cough for 10 days first and then developed fever, vomiting, and diarrhea 3 days before admission. There was no other significant medical history for this child and his family. The parents denied a history of toxic exposure. Upon admission, the body temperature was 36.9°C, heart rate was 109 beats/min, respiratory rate was 27 breaths/min, blood pressure was 92/50 mmHg, oxygen saturation (SpO_2_) was 95–97% on 25% FiO_2_, and the Glasgow coma scale score (GCS) was E1V1M3. His breathing sounds were normal bilaterally on chest auscultation without rales. Babinski sign was positive bilaterally. Other findings from the systemic physical examinations were unremarkable. He was intubated, and intravenous diazepam and midazolam (5 μg/kg/min) were administered, which successfully controlled the recurrent seizure during the first 24 h after admission. Initial arterial blood gas analysis showed pH 7.218, pO_2_ 76 mmHg, pCO_2_ 48.1 mmHg, HCO_3_ 19.6 mmol/L, base excess −8 mmol/L, plasma lactate 4.28 mmol/L, and glucose 15.1 mmol/L. A series of blood tests showed the following results: white blood cell 16.85 × 10^9^/L, hemoglobin 14.4 g/dL, platelets 255 × 10^9^/L, CRP 6.17 mg/L, procalcitonin (PCT) 45 ng/mL, ferritin 8284.1 ng/ml, ammonium 30 μmol/L, transaminases (ALT 478 IU/L, AST 748 IU/L), creatinine 29.6 μmol/L, serum enzymes (CKMB 76 U/L, CK 126 U/L, LDH 1012 U/L), electrolytes (sodium 139.6 mmol/L, potassium 3.79 mmol/L, chloride 106.8 mmol/L, calcium 2.17 mmol/L, and magnesium 0.83 mmol/L), coagulant function (fibrin 1.32 g/L, INR 1.52, D-Dimer 5.56 mg/L). Chest radiograph was normal. Cranial CT showed extensive lesions of low intensity in the white matter, thalamus, brainstem, and cerebellum (Figure 1). Echocardiography showed that cardiac function was normal. The electrocardiograph showed sinus tachycardia. The patient showed rapid clinical deterioration and developed brain stem herniation and shock at hour 7 of admission. Lumbar punctures showed increased intracranial pressure (ICP > 25 mmHg). CSF protein was elevated to 289.2 mg/dL without pleocytosis. CSF virus PCRs were all negative. Cultures of CSF, blood and stool did not identify any pathogens. Electroencephalography (EEG) examination demonstrated diffuse, generalized and slow background activity. Broad-spectrum antibiotics (meropenem and vancomycin) and 20% mannitol (1 g/kg every 6 h) were administered immediately. Multiple organ dysfunction syndrome (MODS) was diagnosed at 17 h after admission. Membrane-based therapeutic plasma exchange was performed 2 times to eliminate proinflammatory mediators in the blood. Methylprednisolone (5 mg/kg every 8 h) and intravenous immunoglobulin (1 g/kg/day × 2 days) were used to reduce hyperinflammation. Considering poor response to treatment and acute clinical worsening, a further toxicological analysis in the blood was performed. The qualitative results of liquid chromatography-mass spectrometry revealed that resmethrin was detected in the blood, confirming the diagnosis of resmethrin poisoning. There is no specific antidote for resmethrin. The child was brain dead at 77 h after admission (Table 1).

**Table 1:** The timeline of events.

**Before admission**	
10 days	Mild cough
3 days	Mild cough, fever, vomiting, diarrhea
11 h	Tremor, fever
12 min	Seizure, depression of consciousness
**After admission**	
5th min	Sedation by intravenous diazepam
3rd h	Recurrent seizure, fever, vomiting, extensive low intensity in the white matter, thalamus, brainstem, and cerebellum on the cranial CT, no shadows on the chest radiograph
7th h	Brain stem herniation, shock, generalized and slow background activity on the EEG
10th h	ICP > 25 mmHg, CSF protein 289.2 mg/dL without pleocytosis
17th h	MODS
35th h	Toxicological analysis
48th h	Patchy high-density shadows on the chest radiograph
77th h	Brain death

## Discussion

Resmethrin can activate sodium channels on neurons and delay their closure, resulting in neuro-hyperexcitability (2). Most patients with pyrethroid poisoning have a suggestive history, such as occupational exposure in adult patients and accidental ingestion in pediatric patients (4, 5). It might be difficult to diagnose pyrethroid poisoning in a timely manner in children without a history of exposure since it has no specific symptoms. The neurotoxic symptoms of pyrethroids included dizziness, headache, hyperexcitation, seizures, and coma (5, 9). It has been reported that seizures and depression of consciousness are related to massive ingestion (4, 10). The history of pyrethroid exposure in our patient was not provided. Moreover, the child showed cough and fever before the symptoms of vomiting and seizures with elevated PCT, which was confusing for the diagnosis of poisoning and oriented the medical team toward bacterial infection. The neurotoxic symptoms, elevated protein in CSF and extensive lesions in the brain indicated severe neuron impairment. Plasma ammonium and ultrasound of the liver were normal in this child without aspirin use, which indicates the impossibility of Reye syndrome. No pathogens, including bacteria, viruses, and fungi, were found in the CSF, excluding infectious encephalitis. The analysis of amino acids and acylcarnitine in the dried blood spot and urine by tandem mass spectrometry was normal, which excluded inherited metabolic diseases. Furthermore, there was no sign of autoimmune diseases.

The cranial CT and CSF analysis in most patients with pyrethroid poisoning were reported to be normal in previous studies (4, 7, 11). MRI might have more advantages than CT in evaluating the severity of neuron impairment. Two previous studies reported abnormal signal intensity of brain MRI in children with pyrethroid poisoning (7, 12). However, MRI was not performed in our study since the child was too ill to tolerate the MRI scan. Surprisingly, extensive lesions of low density were found by cranial CT scan in this child. Abnormality of cranial CT in pyrethroid poisoning was not described in previous studies. This might be explained by the following reasons. First, pyrethroids can prolong sodium current flow, which might induce hyperexcitability and impairment of neurons. The recurrent seizure and elevated protein of CSF in this child indicate the severe neurotoxicity of resmethrin. Second, the child showed recurrent seizures, coma, and elevated ICP quickly, which indicates the possibility of massive ingestion of resmethrin. A limitation is that the concentration of resmethrin in the blood was not evaluated since quantitative toxicological tests were not routinely performed in our hospital. Third, the time lag between resmethrin ingestion and cranial CT might be longer than that in previous studies since the exact time of exposure in our child was not clear.

There is no specific antidote for pyrethroid (9, 11). Treatment against pyrethroid poisoning includes gastric lavage, anti-epilepsy, reduction of intracranial pressure, and organ supportive therapies. In our patient, gastric lavage was not performed since the history of resmethrin exposure was not provided, and it was over 24 h by the time the resmethrin poisoning was confirmed. Recurrent seizures were successfully quickly controlled by intravenous diazepam and midazolam. However, the ICP stayed elevated and the patient developed brain stem herniation even though many therapies were used to reduce intracranial pressure. It was reported that pyrethroids could be quickly detoxified through liver enzyme systems. Resmethrin could be cleared 6–7 days after ingestion (13). No clinical improvement was observed in our child, which might be related to impaired liver function; therefore, detoxification liver functions might have been affected.

In conclusion, we reported a fatal pediatric case of resmethrin poisoning with an extensive cranial CT lesion and elevated CSF protein. This case might extend the knowledge of neuroimaging and CSF analysis with resmethrin poisoning in children.

## Data Availability Statement

The data for this case report are available on request to the corresponding author.

## Ethics Statement

Ethical review and approval were not required for the study on human participants in accordance with the local legislation and institutional requirements. Written informed consent to participate in this study was provided by the participants' legal guardian. Written informed consent was obtained from the legal guardian, for the publication of any potentially identifiable images or data included in this article.

## Author Contributions

LH: study concept and design. DH and DX: acquisition of data. LH and RL: analysis and interpretation of data. LH and SP: drafting of the manuscript. All authors have read and approved the manuscript.

## Funding

This work was supported by the Youth Program of National Natural Science Foundation of China (Grant No. 81902012 to LH). The funders had no role in the design of the study, collection, analysis, and interpretation of data. They had contributed in supporting the publication of this article.

## Conflict of Interest

The authors declare that the research was conducted in the absence of any commercial or financial relationships that could be construed as a potential conflict of interest.

## Publisher's Note

All claims expressed in this article are solely those of the authors and do not necessarily represent those of their affiliated organizations, or those of the publisher, the editors and the reviewers. Any product that may be evaluated in this article, or claim that may be made by its manufacturer, is not guaranteed or endorsed by the publisher.
